# A Unified View of Vibrational Spectroscopy Simulation through Kernel Density Estimations

**DOI:** 10.1021/acs.jpclett.3c00665

**Published:** 2023-04-10

**Authors:** Romain Botella, Andrey A. Kistanov

**Affiliations:** Nano and Molecular Systems Research Unit, University of Oulu, Oulu 90014, Finland

## Abstract

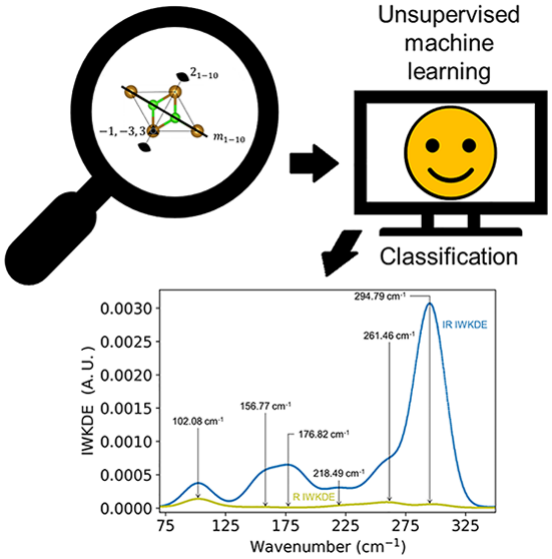

To date, vibrational simulation results constitute more of an experimental support than a predictive tool, as the simulated vibrational modes are discrete due to quantization. This is different from what is obtained experimentally. Here, we propose a way to combine outputs such as the phonon density of states surrogate and peak intensities obtained from ab initio simulations to allow comparison with experimental data by using machine learning. This work is paving the way for using simulated vibrational spectra as a tool to identify materials with defined stoichiometry, enabling the separation of genuine vibrational features of pure phases from morphological and defect-induced signals.

Vibrational properties are fundamental in materials science to ascertaining their dynamic stability^1,2^ and thermodynamic properties.^3^ They also have valuable analytical importance in identifying the materials obtained experimentally and assigning the modes^4,5^ through vibrational spectroscopy. Usually, experimental peaks, whether Raman or infrared (IR), have a spread around the wavenumber of the maximum intensity, the nominal wavenumber, and are usually described as bands.^6−8^ This is well described with the phonon density of states (pDOS) usually analyzed in computational materials science.^9−11^ While well-defined theoretically, it corresponds only to a statistical analysis of the population of energy levels by the different vibrational modes and can be qualitatively compared only to experimental spectra on the absorbance (or transmittance) scale but exhibits accurate wavenumber spread.^12^ To date, these vibrational simulation results constitute more of an experimental support than a fully predictive tool. One of the main issues with this method is that the vibrational modes are theoretically discrete due to the quantization of the vibrations, which is different from what is obtained experimentally,^13^ as mentioned above. This comes from different, nontrivially intertwined types of broadening. Several solutions are used to circumvent this issue such as the use of molecular dynamics and *ab initio*(14−17) or machine learning (ML)^18−20^ to add some of the broadening to the vibrational modes obtained at *T* = 0 K; nonetheless, the supercell method remains widely used.^21−23^ It has the advantage of separating the modes, enabling a precise interpretation of experimental spectra. Using ML to expand simulation methods that are deemed too computationally costly is an interesting path when exploring the material space further. In that case, the use of ML would help to compensate for the drawbacks of costly but more precise simulation methods.^24−26^

Among the different materials to study with IR, two-dimensional (2D) materials have drawn much interest so far because of their interesting properties such as high exciton binding energies and mechanical flexibility.^26−28^ Metal dichlorides (MCl_2_) constitute a family of 2D materials starting to attract interest both computationally^21,29−33^ and experimentally.^34,35^ They are starting to be synthesized experimentally, but their vibrational spectra have never been reported or analyzed to date. They are, therefore, an interesting system for advanced vibrational properties study.

Herein, we present such an implementation of ML and propose a way to combine all of the outputs, such as pDOS and peak intensities obtained with the supercell method (*T* = 0 K), to allow comparison with experimental data by using an unsupervised kernel density estimation (KDE) algorithm. Furthermore, we derive the Cartesian components of each mode, allowing for irreducible representation (irrep) assignment. This result paves the way for the IR/Raman mode separation and subsequent IR simulation that are shown herein. With all of these improvements, we promote the supercell method to the level of truly comparing with experimental data, separating the Raman and IR components of the vibrational modes. Finally, we provide a simulation of the IR spectrum of a compound that has never been explicitly vibrationally characterized before, making this work and this vibrational simulation method a predictive tool rather than just an experimental support.

After simulating the FeCl_2_ monolayer with high accuracy using first-principles simulations, the vibrational modes were computed and subsequently analyzed, as shown in Figure 1. In this figure, it is worth noting that all of the vibrational modes are displayed and used for subsequent calculations. More details on the simulations are shown in Computational Methods and in ref (21). Figure 1a contains the vibrational modes of the FeCl_2_ monolayer represented by Dirac peaks, with heights corresponding to their respective intensities. By comparing the number of distinguishable peaks, which is 23, with the number of degrees of freedom of the entire slab, which is 141, one can see that most of the peaks are actually clusters of modes. This observation shows that the adopted methodology is consistent with both the number of mechanical degrees of freedom^3^ and the nature of the species involved (mass and interatomic constants). Whether the supercell has a size of 1 × 1 × 1 or 10 × 10 × 1, the material is still the same in terms of physicochemical identity. Thus, the distribution of the vibrational modes should be similar or else the experimental identification of materials through vibrational spectroscopy would be more complicated than it actually is experimentally, producing totally different spectra for different material sizes. Therefore, one observed peak in Figure 1a can account for more than one vibrational mode.

**Figure 1:**
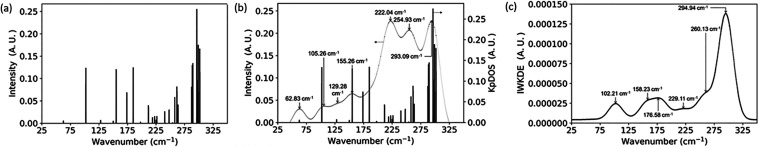
Different representations of all of the vibrational modes of the FeCl_2_ monolayer with (a) a discrete (Dirac peaks) view, (b) a superposition of the discrete view and the KpDOS, and (c) IWKDE. Arrows in (b) and (c) highlight the main signals.

Another way to calculate pDOS is to use the kernel density estimation (KDE) method. KDE is an unsupervised ML algorithm that is mainly used for statistical analysis.^36,37^ It is associated with each point with a kernel function and calculates the entire density profile based on this assumption. We used a Gaussian kernel in this work for availability and experimental accuracy purposes.^38−40^ The results of kernel density estimation of the phonon density of states (KpDOS) along with the Dirac peaks for comparison are shown in Figure 1b. The enlarged image of KpDOS that can be used as a surrogate for PDOS is shown in Figure S1.

Seven bands are observed, the highest KpDOS values are associated with the wavenumbers 222.04, 254.93, and 293.09 cm^–1^, and the other bands are more than 4 times less than the highest KpDOS bands. The use of the KDE algorithm gives a continuous curve from a discrete collection of points, which is observed in pDOS. The large width of the bands is enables a smooth distribution, which is generally not noticed in classical pDOS^8−10^ but is often obtained in experimental work.^41,42^ The width can be modified to enable better flexibility in comparison with the experimental results. As can be seen in Figure S2, the width is controlling the number of bands visible on the KpDOS. The distribution becomes similar to more usual pDOS when the width decreases (e.g., 0.5 cm^–1^), and a potato-shaped band is obtained when the width increases (e.g., 18 cm^–1^).

However, in experimental work, the population of the energy values is not the only thing observed. According to the Beer–Lambert law, for a concentration of 1 mol·L^–1^ and a path length of 1 cm, the distribution of the extinction coefficient is due to both the population of the energy levels and the values (intensities). While pDOS is well-defined theoretically, it is not clear how to include this term theoretically. On the other hand, the KDE algorithm can calculate the weighted density of a data collection.^37^ Provided the weights are equal to the intensity of the vibrational modes, the intensity-weighted kernel density estimation (IWKDE) spectrum can be computed, as shown in Figure 1c.

We can see from Figure 1c that a new set of six bands are obtained. The wavenumbers of the bands are very different from those observed in KpDOS. The highest-intensity band has wavenumber of 294.94 cm^–1^, and the other bands have the same IWKDE ratio as in KpDOS. The morphology of the IWKDE spectrum is not identical to the KpDOS profile, which is also one drawback observed when comparing pDOS or KpDOS and experimental vibrational spectra.

The apparent differences can be rationalized through Figure 1b, showing the superposition of the Dirac intensities with KpDOS. We can see that the KpDOS band at ca. 62 cm^–1^ disappears because of its very small intensity as well as the KpDOS bands at ca. 222 and 255 cm^–1^ that are greatly reduced because of the large difference between intensities and KpDOS values. These discrepancies could explain the observation of certain peaks in experimental work, as others are not seen in the expected wavenumber range. By adding this feature, the results reported here are one step closer to accurate vibrational spectrum simulation only from the output of a well-known method, by unifying pDOS and discrete Dirac peak views.

Usually, the vibrational modes are designated by the irreps to which they belong. This sorting is performed through normal-mode analysis (NMA) using group theory.^43^ The naming of the different modes is nontrivial, forcing the use of more straightforward approaches^7^ in the absence of a clear guide for attribution. As experimentalists use polarization to assign bands to group theory predictions, Cartesian components of the IR intensities can also be calculated (eqs 9–11),^44^ enabling the assignment of the different vibrational modes to their respective irreps. By doing so, simulated vibrational spectra of materials not yet synthesized are becoming utterly predictive rather than a simple support for experimental endeavors.

According to group theory, the FeCl_2_ monolayer belongs to the *P*3̅*m*1 space group (number 164) with the symmetry operations shown in Figure 2a for its 1 × 1 × 1 unit cell. The six optical modes of this cell can be decomposed into irreps as follows
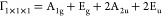
1with A_1g_, A_2u_, E_g_, and E_u_ irreps of the *P*3̅*m*1 space group associated with the different vibrational modes of FeCl_2_. E modes are in-plane vibrations, A modes are out-of-plane, g are centrosymmetric modes, and u are noncentrosymmetric modes. More details on group theory can be found in ref (43).

**Figure 2:**
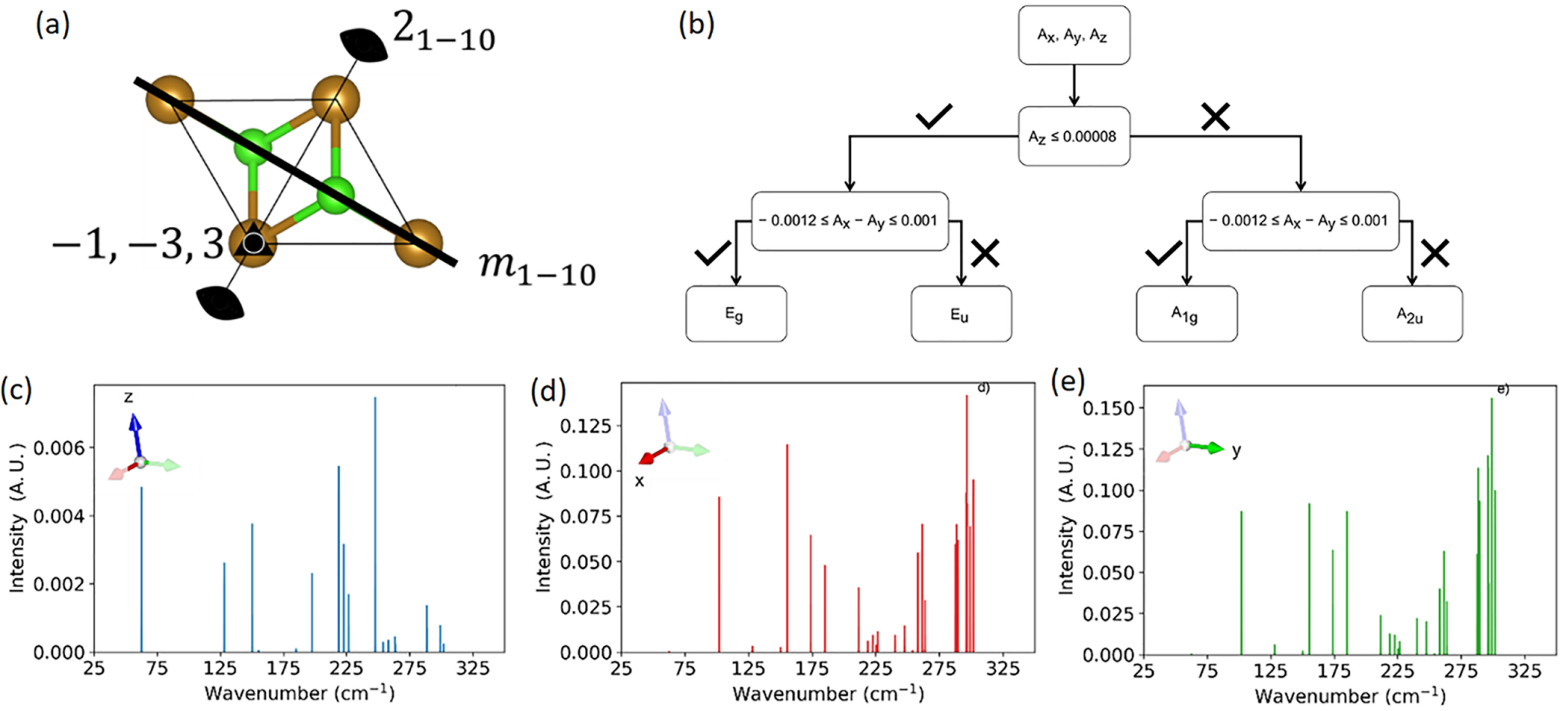
(a) Representation of the FeCl_2_ monolayer unit cell and its symmetry operations, (b) decision tree diagram for the classification of the modes in the different irreps, (c) *z*-polarized discrete (Dirac) intensities (blue) of the modes of the FeCl_2_ monolayer, (d) *x*-polarized discrete (Dirac) intensities (red) of the modes of the FeCl_2_ monolayer, and (e) *y*-polarized discrete (Dirac) intensities (green) of the modes of the FeCl_2_ monolayer. The nomenclature is from ref (46).

However, the supercell used in this work consists of 4 × 4 × 1 unit cells. The 141 optical modes associated with such a cell can also be decomposed in irreps according to
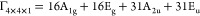
2

From the character of Table S1, E and A differ in their orientations: E modes are in-plane (*z* component = 0) and A modes are out-of-plane. While group theory is very clear about the orientation of the atomic displacements, the results of the *ab initio* calculations clearly show a deviation from the theory, as the only way to achieve the irrep decomposition in eq 2 is to set a threshold (Figure 2b) on the Cartesian components’ values. This deviation comes from many factors related to the approximations made in the calculations such as the anharmonicity of the atomic displacements, the strain applied to the cell upon optimization, the nonideal position of the atoms in the as-calculated cell, and the supercell size. As NMA is based on the harmonic approximation, many works have been able to calculate the degree of anharmonicity^45^ in the vibrational spectra simulated. While this result is clearly visible in the frequency calculation, the true deviation comes from the shape of the potential energy surface. Hence, a threshold is needed to assign their irrep properly.

After the first classification step, 94 E (E_g_+ E_u_) modes and 47 A (A_1g_+ A_2u_) modes are obtained. The second step is to classify the modes between g (E_g_, A_1g_) and u (E_u_, A_2u_) irreps. From the character table (Table S1) of the D_3d_ group, the A_1g_ irrep corresponds to the fully symmetric mode, hence the signal should be unchanged by any symmetry operation. That means that the *x* and *y* components should be equal due to the threshold applied while the *z* component is different from *x* and *y*, and a threshold is applied for the same reasons as discussed above (Figure 2b). The same holds true for E_g_. Form this, the classification in eq 2 is obtained, along with the detailed attribution and values of the *x* (I_*x*_), *y* (I_*y*_), and *z* (I_*z*_) components (Figure 2c–e). It is worth noting that the minimum value of the threshold needed to classify properly is presented in Figure 2b. Most of the modes were already assigned for thresholds below the values given here, nuancing somehow the shift from the harmonic predictions.

Following the irrep assignment, the different modes can be separated into Raman-active (E_g_+ A_1g_) and IR-active (E_u_+ A_2u_) modes, as shown in Figure 3a–c. As noticed in Figure 1, the number of bands is 6 (Figure 3c), which is smaller than the number of modes, and the bands are not necessarily centered at the same wavenumbers. Regarding the IWKDE spectrum (Figure 3c), the wavenumbers and relative intensities of the bands are also changing. The highest band has wavenumber 294.79 cm^–1^ in the IR IWKDE. Regarding the wavenumbers of the bands, they do not vary significantly for most of them, which can be interpreted as the same mode being active in both kinds of spectroscopy.

**Figure 3:**
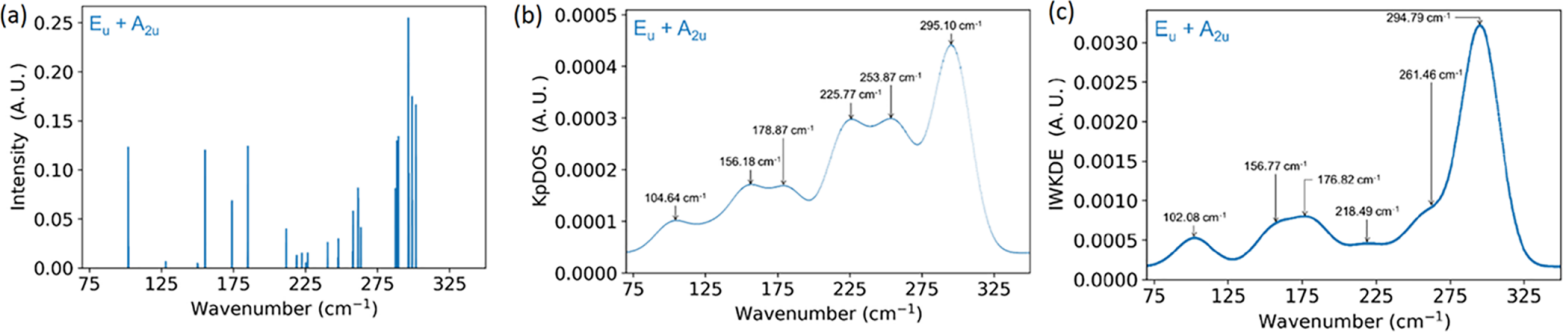
Different representations of the IR-active vibrational modes of the FeCl_2_ monolayer with (a) a discrete (Dirac peaks) view, (b) the KpDOS, and (c) the IWKDE. Arrows in (b) and (c) highlight the main signals.

The IWKDE spectrum and the Dirac peaks (discrete modes), presented in Figure 4, are superposed for the IR. Despite a strong resemblance of Figure 4c with Figure 1c, only the IR-active modes are displayed, hence the use of a different color. From this, one can see that the simulated bands ignore several modes that have smaller intensities but still contain information. These results recommend caution as to the quick and easy assignment of a complex system’s spectroscopic results without well-established justifications. While it is true that bands can hide several minor signals and therefore hide some information, it is still true that the most intense bands are expressed in the spectrum, enabling us to obtain the main information about the vibrational state of the sample. Figure 4a, along with Table 1, paves the way for the irrep assignment of the different bands observed in the simulated band spectra.

**Figure 4:**
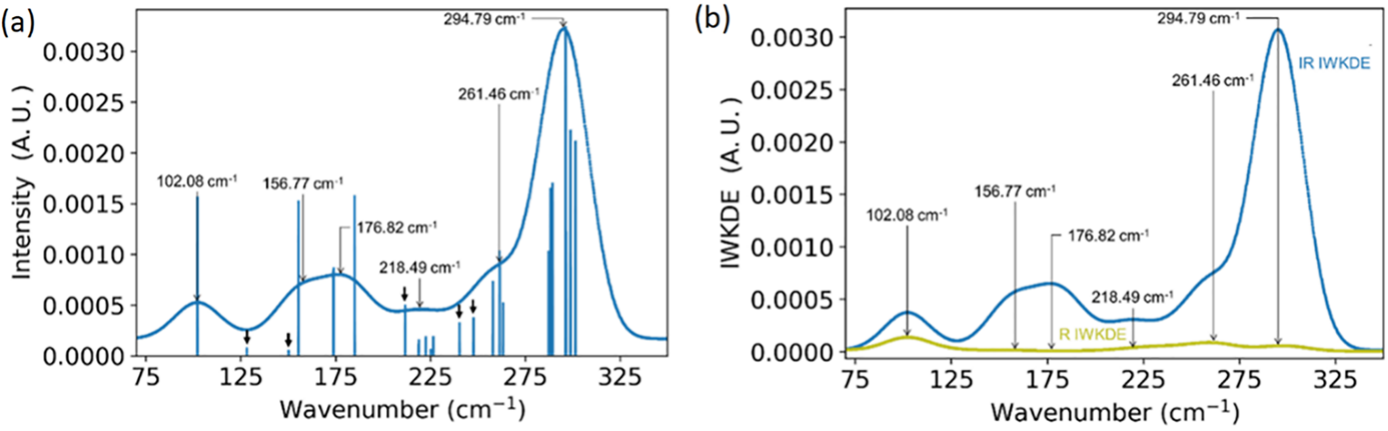
Superposition of (a) the IR Dirac modes, the associated IWKDE spectrum, and the associated IWKDE spectrum and (b) IR- and Raman-active mode IR IWKDE spectra. Arrows highlight the main bands, and the Dirac intensity peaks have been normalized to be compared with IWKDE spectra.

**Table 1:** Distribution of the Contribution of the Different irreps to the IWKDE IR^a^

	Infrared	
Wavenumber (cm^–1^)	E_u_	A_2u_
102.06	100%	0%
156.77	62%	38%
176.22	100%	0%
218.49	75%	35%
261.46	58%	42%
294.79	69%	31%

a See Computational Methods.

As D_3d_ is centrosymmetric, Raman and IR modes should be complementary (i.e., the IR intensity of Raman-active modes should be zero). To validate this theoretical conclusion, the IR intensities of the IR-active modes and the Raman-active modes have been computed separately and superposed in Figure 4b. While the comparison of the IWKDE for the IR and Raman bands (Figure 4b) shows a clear difference in intensity between both simulated spectra, it is worth noticing that the IR intensities of Raman-active bands overlap. The high intensity of IR-active modes with respect to the IR intensity of Raman-active modes can be explained by the high ionicity of the FeCl_2_ monolayer samples, proposed in previous work.^21^ As a consequence, the dipole moment upon vibration is much more likely to change than the polarizability. The IR activity coefficient is therefore going to be much stronger than the Raman activity coefficient, explaining the difference in activity and cross-validating the sorting methodology herein. This observation also explains the resemblance between the IWKDE shown in Figure 1c and the IR IWKDE in Figure 4a.

From this result, it can also be recommended to use IR to obtain a much stronger signal, although the information may be mixed, as the bands are less pure with respect to the irrep attribution. This can be observed experimentally and can be interpreted as a deviation from ideality, as the mode is being somewhat activated by a sample not ideally prepared. Here it is clearly shown that the Raman-active and IR-active modes can have an overlapping band to a certain degree despite the theoretical rule of them being incompatible. This possibly comes from the anharmonicity mentioned earlier. This comparison is possible only because of the irrep attribution of the different mode and, to the best of our knowledge, is the first of this kind in the literature.

As not all of the bands are purely assigned to one irrep, they should output mixed information and are therefore less trustworthy than pure ones without more advanced spectroscopic methods to selectively analyze one irrep or the other. The IR band at ca. 261.46 cm^–1^ is a mixture of modes from E_u_ (58%) and A_2u_ (42%) irreps. It has the highest degree of mixing and, as a consequence, is the least reliable. On the other hand, the 176.22 cm^–1^ band belongs entirely to E_u_ irrep, which makes it one of the most trustworthy bands in the IR spectrum. This work also gives insights into how to assess the reliability of band evolution (inverse trends due to different species), based on computational results.

In this work, the FeCl_2_ monolayer vibrational spectra have been simulated and studied in the spectroscopic framework. An unsupervised ML KDE algorithm has been used to unify pDOS and intensity, getting a spectrum for the first time with this method with a realistic appearance and allowing for a consistent comparison of experimental data. Moreover, simulation does not suffer from the drawbacks of experimental synthesis (e.g., impurities, overtones, and nonlinear baselines) and therefore enables the separation of the true spectroscopic signal of the desired material from the above-mentioned experimental spectroscopic perturbations. It will also provide a way to estimate the presence of defects, stoichiometry variations, and other modifications of the material, enabling performance improvement quantitatively, provided that the right simulation outputs are given.

## Computational Methods

The FeCl_2_ monolayer was geometrically optimized, and its stability was checked in previous work.^21^ The geometry of the primitive unit cell of the FeCl_2_ monolayer is also available in the 2DMatPedia database (ID dm-3574).^47^ To ensure the reliability of the model, we also performed geometry optimization for the here-considered FeCl_2_ monolayer primitive unit cell and the 4 × 4 × 1 supercell. The FeCl_2_ monolayer stabilizes in a trigonal lattice having lattice parameters of *a* = *b* = 3.41 Å, which is in good agreement with previous works reporting on the FeCl_2_ monolayer.^21^ The geometry optimization was conducted using the plane-wave method as implemented in the Vienna Ab Initio Simulation Package (VASP). The vibrational modes were computed for the 4 × 4 × 1 supercell using the finite difference method as implemented in VASP. Zone-center (Γ-point) frequencies were calculated. For all calculations, the Perdew–Burke–Ernzerhof exchange-correlation functional under the generalized gradient approximation was utilized.^48^ The periodic boundary conditions were applied for the two in-plane transverse directions, while a vacuum space of 20 Å was introduced in the direction perpendicular to the surface plane.

The ML part was performed using the sci-kit learn package.^49^ The KDE algorithm associates each point with a kernel function. Mathematically, it can be presented as follows:
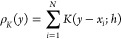
3With ρ_K_(*y*), the density estimate was calculated with the kernel function *K* for a collection of *N* points *x*_*i*_ at value *y*. This algorithm includes the possibility to weight each data point as follows
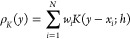
4with *w*_*i*_, the weight of point *x*_*i*_, enabling the combination of the distribution of the vibrational modes and their respective intensity, as will be discussed below.

To smooth the KDE profile obtained, the density of points throughout the wavenumber range must be increased to avoid having a profile resembling a discrete one. To this end, a baseline has been manually added to the wavenumber range of −22.5 to 472.5 cm^–1^. The baseline is constituted of points spaced from 0.225 cm^–1^. The value is not central, as the curves obtained have the same shapes but different intensities.

The Cartesian components of the intensity can be separated from the nonpolarized outputs of the supercell methods in a rigorous manner. The absorbance is defined by
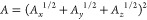
5with *A_i_* (*i* = *x*, *y*, *z*) being the Cartesian components of the absorbance. After development, we obtain
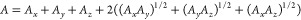
6

On the other hand, the intensities of the vibrational modes calculated with the supercell method are defined by
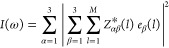
7with *I*(ω) being the intensity of the spectrum, α and β being any of the Cartesian coordinates, *l* being an ion in the system containing *M* ions, *Z*_αβ_^*^(l) being the Born effective charge tensor for the *l*th ion in the α, β directions, and *e*_β_(*l*) being the β component of the ionic displacement of the *l*th ion. After the development of eq 7, eq 8 is obtained
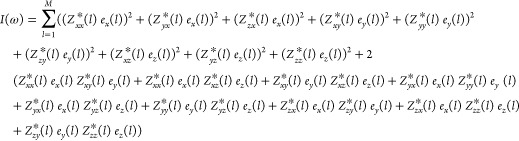
8exhibiting nine squared terms also present in the following nine terms. By identification, grouping the terms having the same displacement vector, we obtain eqs 9–11
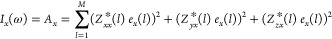
9
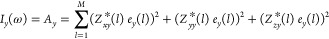
10
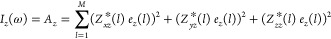
11with *I_i_*(ω) (*i* = *x*, *y*, *z*) being the Cartesian components of the intensity. The threshold for the classification of the vibrational modes is set to be the minimum values of *A*_*x*_, *A*_*y*_, and *A*_*z*_ allowing the correct number of modes in each irrep.

The band irreducible representation (irrep) percentage (in numbers) of modes in the different bands is calculated according to
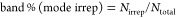
12where *N*_irrep_ is the number of modes belonging to irrep that are included in the band observed in the IKWDE spectrum and *N*_total_ is the total number of modes included in the band.

For visualization of the results, the matplotlib was used.^50^

## Supporting Information Available

Kernel density estimated phonon density of states (Figure S1); KpDOS profiles for the vibrational modes of FeCl_2_ for different bandwidths (Figure S2); and wavenumbers, Cartesian polarized intensities, and corresponding irreps for the 4 × 4 × 1 supercell of the FeCl_2_ monolayer (Table S1) (PDF)
