# Supplementary material for: A Unified View of Vibrational Spectroscopy Simulation through Kernel Density Estimations
Source: J Phys Chem Lett. 2023 Apr 10;14(15):3691–7. doi: 10.1021/acs.jpclett.3c00665 (PMC10123815; doi:10.1021/acs.jpclett.3c00665)
Supplement: Supplementary file 1 — jz3c00665_si_001.pdf [file jz3c00665_si_001.pdf]

Supporting Information for

A Unified View of Vibrational Spectroscopy Simulation through Kernel Density Estimation

Romain Botella* and Andrey A. Kistanov

Nano and Molecular Systems Research Unit, University of Oulu, Oulu 90014, Finland

*Corresponding author: romain.botella@oulu.fi

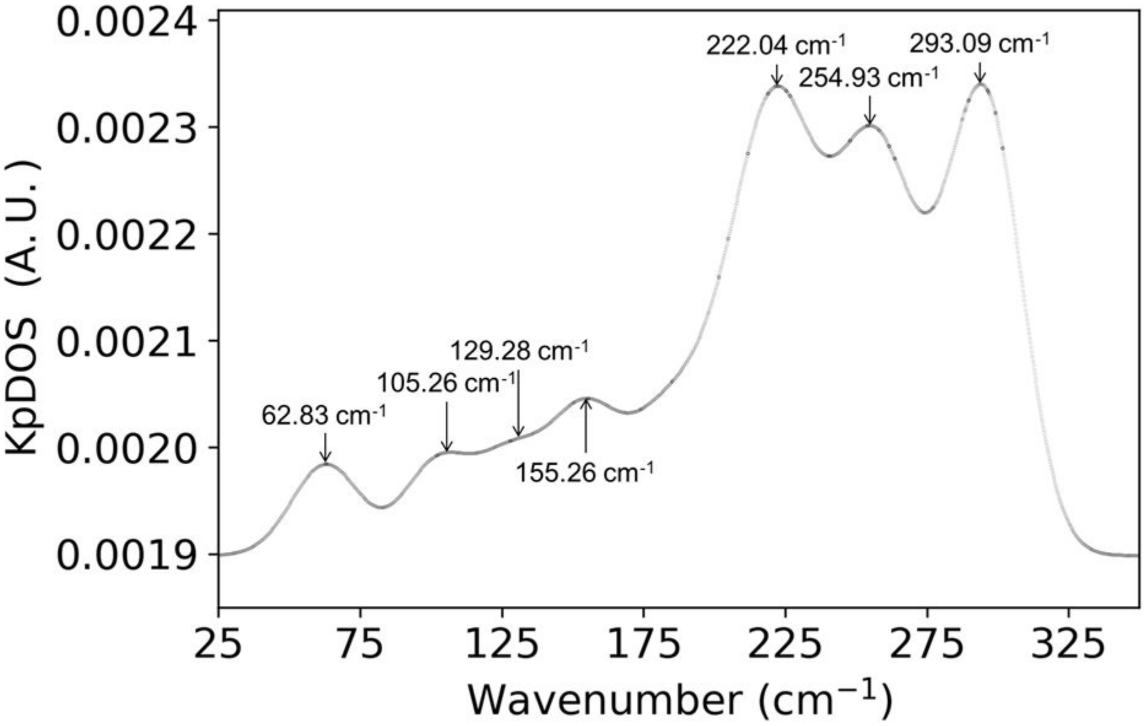

Figure S1. Kernel density estimated phonon density of states (KpDOS).

Table S1. Wavenumbers, cartesian polarized intensities and corresponding irreps for the 4×4×1 supercell of the $FeCl_2$ monolayer.

number	Wavenumber (cm^{-1})	I_x	I_y	I_z	Irrep.	number	Wavenumber (cm^{-1})	I_x	I_y	I_z	Irrep.
1	301.733	0.09477	0.01680	0.00022	A_{2u}	71	226.567	0.00564	0.00059	0.00001	E_u
2	301.711	0.00182	0.00000	0.00000	E_u	72	226.565	0.01093	0.00388	0.00000	E_u
3	301.676	0.01377	0.09952	0.00022	A_{2u}	73	226.559	0.00034	0.00400	0.00000	E_u
4	301.667	0.00118	0.00446	0.00000	E_u	74	226.546	0.00380	0.00760	0.00074	A_{2u}
5	301.659	0.00536	0.00237	0.00000	E_u	75	226.543	0.00060	0.00512	0.00167	A_{2u}
6	301.650	0.08662	0.07886	0.00001	A_{2u}	76	225.196	0.00363	0.00336	0.00000	E_g
7	299.131	0.06056	0.00766	0.00017	A_{2u}	77	225.189	0.00000	0.00000	0.00000	E_g
8	299.094	0.06898	0.00951	0.00014	A_{2u}	78	225.175	0.00044	0.00045	0.00000	E_g
9	299.078	0.03456	0.02407	0.00075	A_{2u}	79	225.165	0.00322	0.00177	0.00000	E_u
10	299.073	0.01063	0.00280	0.00000	E_u	80	225.157	0.00015	0.00023	0.00000	E_g
11	299.069	0.01859	0.15573	0.00000	E_u	81	225.149	0.00001	0.00179	0.00000	E_u
12	299.060	0.01370	0.01340	0.00012	A_{1g}	82	222.598	0.00346	0.01122	0.00001	E_u
13	296.887	0.08139	0.01448	0.00000	E_u	83	222.571	0.00238	0.00178	0.00315	A_{1g}
14	296.850	0.00000	0.04280	0.00000	E_u	84	222.542	0.00901	0.00200	0.00116	A_{2u}
15	296.788	0.03173	0.03863	0.00000	E_u	85	221.698	0.00000	0.00000	0.00000	E_g
16	296.443	0.01157	0.00104	0.00000	E_u	86	221.656	0.00000	0.00000	0.00000	E_g
17	296.438	0.00938	0.02209	0.00000	E_u	87	218.908	0.00000	0.01232	0.00000	E_u
18	296.422	0.00042	0.00405	0.00000	E_u	88	218.884	0.00537	0.00037	0.00000	E_u
19	296.402	0.14151	0.11306	0.00000	E_u	89	218.858	0.00000	0.00008	0.00046	A_{1g}
20	296.364	0.08737	0.12071	0.00000	E_u	90	218.837	0.00566	0.00001	0.00352	A_{2u}
21	296.346	0.00481	0.00742	0.00000	E_u	91	218.809	0.00105	0.00008	0.00543	A_{2u}
22	289.682	0.06090	0.00020	0.00000	E_u	92	218.789	0.00081	0.00004	0.00464	A_{2u}
23	289.678	0.06140	0.00447	0.00000	E_u	93	211.740	0.01320	0.02137	0.00000	E_u
24	289.670	0.00267	0.00242	0.00000	E_g	94	211.733	0.00001	0.00117	0.00000	E_g
25	289.664	0.04068	0.09294	0.00000	E_u	95	211.728	0.00106	0.02341	0.00000	E_u
26	289.640	0.04670	0.07155	0.00000	E_u	96	211.718	0.03523	0.00371	0.00000	E_u
27	289.634	0.00070	0.03632	0.00000	E_u	97	211.706	0.00000	0.00013	0.00000	E_g

28	288.554	0.06995	0.02849	0.00068	A_{2u}	98	211.671	0.00022	0.00001	0.00000	E_g
29	288.432	0.05007	0.00000	0.00135	A_{2u}	99	204.849	0.00000	0.00000	0.00000	E_g
30	288.413	0.01585	0.11317	0.00016	A_{2u}	100	204.826	0.00000	0.00000	0.00000	E_g
31	287.494	0.05933	0.01779	0.00000	E_u	101	204.795	0.00000	0.00000	0.00000	E_g
32	287.405	0.01979	0.06068	0.00000	E_u	102	201.513	0.00000	0.00000	0.00000	E_g
33	277.096	0.00000	0.00000	0.00000	E_g	103	201.482	0.00000	0.00000	0.00000	E_g
34	277.085	0.00000	0.00000	0.00000	E_g	104	201.475	0.00000	0.00000	0.00000	E_g
35	277.078	0.00000	0.00000	0.00000	E_g	105	197.780	0.00000	0.00000	0.00228	A_{1g}
36	276.308	0.00000	0.00000	0.00000	E_g	106	185.061	0.01227	0.00220	0.00000	E_u
37	263.611	0.00104	0.00069	0.00000	E_g	107	185.056	0.00229	0.00696	0.00001	E_u
38	263.593	0.02810	0.00428	0.00010	A_{2u}	108	185.051	0.03656	0.0868	0.00000	E_u
39	263.583	0.00028	0.00046	0.00000	E_g	109	185.037	0.00878	0.00152	0.00000	E_u
40	263.566	0.00325	0.00512	0.00022	A_{2u}	110	185.022	0.02521	0.00017	0.00000	E_u
41	263.561	0.00886	0.03194	0.00000	E_u	111	185.009	0.04771	0.03578	0.00000	E_u
42	263.505	0.00096	0.00004	0.00044	A_{2u}	112	173.932	0.02614	0.02981	0.00000	E_u
43	261.635	0.07031	0.00004	0.00000	E_u	113	173.902	0.00477	0.06330	0.00000	E_u
44	261.620	0.00163	0.00011	0.00000	E_u	114	173.859	0.06406	0.00174	0.00000	E_u
45	261.590	0.00619	0.04067	0.00000	E_u	115	155.328	0.00305	0.00309	0.00000	E_g
46	261.587	0.02028	0.02030	0.00000	E_g	116	155.313	0.11410	0.00551	0.00000	E_u
47	261.567	0.01817	0.06256	0.00000	E_u	117	155.304	0.00513	0.00859	0.00000	E_u
48	261.536	0.00734	0.00009	0.00000	E_u	118	155.303	0.02882	0.02768	0.00000	E_u
49	258.052	0.00236	0.03960	0.00012	A_{2u}	119	155.253	0.00064	0.01686	0.00000	E_u
50	258.033	0.05433	0.00284	0.00000	E_u	120	155.238	0.00110	0.09134	0.00000	E_u
51	257.978	0.00054	0.01525	0.00033	A_{2u}	121	150.212	0.00242	0.00028	0.00014	A_{2u}
52	253.965	0.00000	0.00000	0.00022	A_{1g}	122	150.175	0.00035	0.00188	0.00103	A_{2u}
53	253.959	0.00038	0.00015	0.00015	A_{1g}	123	150.163	0.00000	0.00065	0.00374	A_{2u}
54	253.944	0.00032	0.00011	0.00018	A_{1g}	124	128.006	0.00035	0.00005	0.00021	A_{1g}
55	253.937	0.00000	0.00010	0.00000	E_g	125	127.988	0.00220	0.00054	0.00260	A_{2u}
56	253.929	0.00000	0.00015	0.00000	E_g	126	127.961	0.00000	0.00010	0.00195	A_{1g}
57	253.925	0.00000	0.00009	0.00026	A_{1g}	127	127.955	0.00300	0.00010	0.00079	A_{2u}

58	247.854	0.01423	0.01339	0.00182	A_{2u}	128	127.948	0.00107	0.00083	0.00216	A_{1g}
59	247.811	0.00298	0.00079	0.00012	A_{2u}	129	127.940	0.00050	0.00548	0.00001	A_{2u}
60	247.781	0.00728	0.01960	0.00029	A_{2u}	130	102.025	0.00663	0.01401	0.00000	E_u
61	247.767	0.00656	0.00072	0.00001	E_u	131	102.013	0.03612	0.08675	0.00000	E_u
62	247.753	0.00227	0.00091	0.00255	A_{2u}	132	102.010	0.03814	0.03829	0.00000	E_g
63	247.731	0.00239	0.00012	0.00746	A_{2u}	133	102.001	0.00094	0.02573	0.00000	E_u
64	240.402	0.00061	0.00007	0.00000	E_g	134	101.990	0.00096	0.00154	0.00000	E_g
65	240.385	0.00373	0.02164	0.00000	E_u	135	101.972	0.08508	0.00165	0.00000	E_u
66	240.364	0.00887	0.00845	0.00000	E_g	136	62.918	0.00000	0.00000	0.00124	A_{1g}
67	240.360	0.0090	0.00130	0.00000	E_u	137	62.897	0.00000	0.00000	0.00061	A_{1g}
68	240.349	0.00634	0.0020	0.00000	E_u	138	62.833	0.00000	0.00000	0.00482	A_{1g}
69	240.349	0.00488	0.00002	0.00000	E_u	139	62.824	0.00000	0.00002	0.00073	A_{1g}
70	226.578	0.00000	0.00010	0.00000	E_g	140	62.763	0.00000	0.00003	0.00044	A_{1g}
						141	62.747	0.00000	0.00006	0.00000	E_g

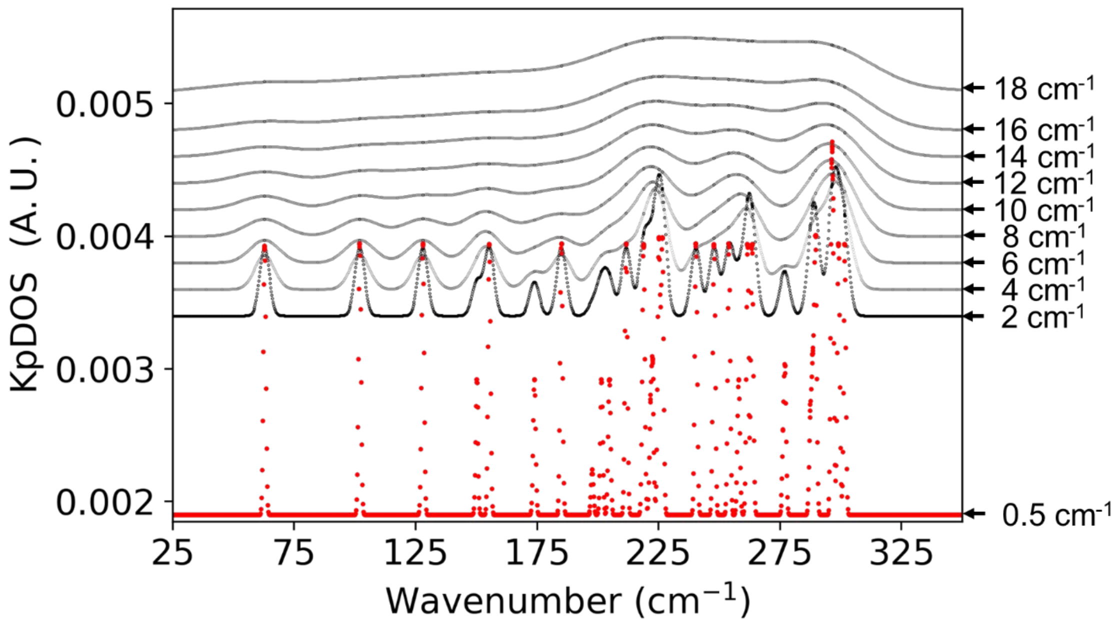

Figure S2. KpDOS profiles for the vibrational modes of $FeCl_2$ for different bandwidth. For 0.5 cm^{-1} bandwidth, the profile is colored in red for visibility purposes. The other plots in the main text are made using a 12 cm^{-1} bandwidth.
